# Novel pre-vascularized tissue-engineered dermis based on stem cell sheet technique used for dermis-defect healing

**DOI:** 10.1093/rb/rbaa039

**Published:** 2020-10-08

**Authors:** Zengjie Fan, Xuzhuzi Xie, Shengqian Zhu, Xiaozhu Liao, Zhengrong Yin, Yujue Zhang, Fengzhen Liu

**Affiliations:** 1 School of Stomatology, Lanzhou University, Donggang West Road 199, Gansu 730000, People’s Republic of China; 2 Liaocheng People’s Hospital, Medical College of Liaocheng University, Liaocheng 252000, People’s Republic of China

**Keywords:** bone marrow mesenchymal stem cell sheet, fibroblasts cell sheet, endothelial cells, pre-vascularized tissue-engineered dermis, dermis-defect repair

## Abstract

Insufficient donor dermis and the shortage of three-dimensional vascular networks are the main limitations in the tissue-engineered dermis (TED). To solve these problems, we initially constructed pre-vascularized bone marrow mesenchymal stem cell sheet (PBMCS) and pre-vascularized fibroblasts cell sheet (PFCS) by cell sheet technology, and then superimposed or folded them together to construct a pre-vascularized TED (PTED), aiming to mimic the real dermis structure. The constructed PTED was implanted in nude mice dorsal dermis-defect wound and the wound-healing effect was quantified at Days 1, 7 and 14 via the methods of histochemistry and immunohistochemistry. The results showed that PTED could rapidly promote the wound closure, especially at Day 14, and the wound-healing rate of three-layer PTED could reach 97.2% (*P* < 0.01), which was faster than the blank control group (89.1%), PBMCS (92.4%), PFCS (93.8%) and six-layer PTED (92.3%). In addition, the vessel density in the PTED group was higher than the other groups on the 14th day. Taken together, it is proved that the PTED, especially three-layer PTED, is more conducive to the full-thickness dermis-defect repair and the construction of the three-dimensional vascular networks, indicating its potential application in dermis-defect repair.

## Introduction

As an essential natural barrier organ of human beings, the skin has functions of resisting stimuli in the external environment (e.g. physical, chemical and biological) and preventing the dehydration of the body [1]. The epidermis of skin holds immense promise for the keratinocyte layer regeneration, which is mainly dependent on the dermis. The dermis, rich in collagen, plays a crucial role in retaining the functionality for full-thickness skin and supporting well-vasculature [2]. Severe skin-deep injuries involved in the dermis, such as extensive burns, soft tissue trauma and necrotizing skin disease, can significantly affect the quality of life of patients and increase their financial burden. The deep dermis defects are often haunted by scar contracture in the process of wound healing, stumbling the demonstration of aesthetics and the presentation of the function. The transplanted skin, however, has less mechanical support from the dermis and is unable to resist friction, thus giving rise to blistering, ulceration and infection easily. More importantly, skin graft usually ends up with necrosis owing to the insufficient blood supply to the dermis caused by a lack of blood vessels. Therefore, the construction of tissue-engineered dermis (TED) has gained increasing interest as a promising method in the repair, regeneration and functional reconstruction of skin tissue.

Nowadays, although autologous skin transplantation is the gold standard for the treatment, there also exist problems such as insufficient donors [3]. In 1975, it was demonstrated for the first time that autologous epithelial cells could be isolated and cultured [4], which laid a specific foundation for subsequent tissue engineering skin. To date, TED mainly consists of synthetic dermis substitute and natural decellularized dermal matrix. With natural skin characteristics, synthetic skin substitute combined with epidermal and dermis cells themselves or with biological scaffolds is highly anticipated [5]. Among them, the dermis regeneration scaffold needs certain traits. For example, the existing research support materials with natural materials, synthetic materials, biodegradable materials and so on are like commercially available synthetic dermal substitute: Integra and Dermagraft [6, 7], but they are often derived by sources outside the body’s immune rejection and are characterized by its low-cell biocompatibility and cell utilization and lower vaccination density, and uneven distribution. At the same time, because adverse vascularization involves cell necrosis in the centre of synthetic dermal substitutes, it is difficult to form a three-dimensional (3D) full-thickness skin structure [8]. For the natural decellularized dermal matrix, deficient cells raise difficulties in skin graft survival, wound-healing cycle and vascularization. Therefore, it is urgent to find a new method which can not only meet the donor shortage of TED, but also can improve the vascularization.

In the past few decades, cell-based technology, especially applications of cultivated bone marrow mesenchymal stem cells (BMSCs) have also been presented to exhibit some superiorities for the study of skin repair. In BMSCs, there exist certain differentiations among functions, ability to repair itself and update [9], the secretion of ECM to construct cells, the micro-environment needed for cell proliferation and adhesion [10] and conduciveness to the formation of the vascular system [11]. Also, numerous literature has strongly identified that BMSCs genetically modified by green fluorescent protein are transplanted to skin defects. It is found that BMSCs are the vital contributors to skin repair, and can also differentiate into some skin keratinocytes and sebaceous glands [12–14]. Unfortunately, most of the traditional methods of BMSC transplantation (e.g. directly injected suspended cell fluid) may result in a significant loss in the functionality and integrity of BMSCs.

In light of the above developments, cell sheet technology (CST) is a technology that physically separates the amplified and fused cells from the bottom of the culture dish to obtain the cell sheet structure [15]. This technique relies only on the ECM derived from the cells as the scaffold material and the cells themselves are regarded as the seed cells. Hence, the structure of the cell sheet constructed is similar to healthy human tissues [16]. The advantage of the cell sheet is to avoid immune rejection caused by the implantation of existing exogenous tissue dermis into the human body [17]. In addition, the cell sheet contains cells that can be directly involved in the rehabilitation and reconstruction of the organization, thus improving the wound-healing rate. Multiple pieces of research confirm that the cell sheet has a useful application in the field of cornea, periodontal ligament, bladder epithelium, esophageal epithelium, myocardium and liver repair [17–19]. However, based on the tissue-engineered CST, vascularization is considered to be crucial in dermal tissue homeostasis and repair. The existing research has adopted some cultivation of endothelial cells (ECs) and BMSCs cell sheet (BMCS) for dermis repair progress. For example, Radke et al. [20] used a pre-vascularized stem cell sheet to repair full-thickness skin damage; however, the wound-healing process was relatively slow and it did not reflect the characteristics of dermal repair. However, recent studies have rarely implemented co-cultured BMSCs, ECs and fibroblasts (Fb) to form the cell sheets and mimic the real dermis structure. There have been few studies on the 3D cultured system based on the 3D cell sheets.

Therefore, in this study, CST could be used to build pre-vascularized BMCS (PBMCS) and pre-vascularized Fb cell sheet (PFCS), respectively. Finally, we aim to construct the pre-vascularized tissue-engineered dermis (PTED) by superimposing or folding them together. This study is expected to obtain a new type of multifunctional PTED with dermis structure, to address the obstacles of the existing tissue dermis-defects and to mimic the development of new treatment for the repairing and healing of the deep dermis-defect wounds.

## Materials and methods

### Materials

DMEM/F12, DMEM/high glucose, phosphate-buffered saline (PBS), penicillin–streptomycin, l-glutamine and trypsin/EDTA were purchased from HyClone Laboratories Inc. (UT, USA). The culture medium of ECs was purchased from iCell Bioscience Inc. (Shanghai, China). Fetal bovine serum (FBS) was purchased from SeraPro Inc. (Beijing, China). Rabbit anti-CD31 antibody was purchased from Bioss & Technology Co., Ltd. (Beijing, China). Donkey anti-mouse antibody was purchased from Abcam Inc. (Shanghai, China). The DAPI was purchased from Meilun Biotechnology Co., Ltd. (Dalian, China). Chloral hydrate and bovine albumin (BSA) were purchased from Solarbio Science & Technology Co., Ltd. (Beijing, China). Triton X-100 and ascorbic acid were purchased from Sigma-Aldrich Co., Ltd. (MO, USA).

### Cells isolation and culture

The BMSCs were harvested from the Chinese big ear white rabbit obtained from Lanzhou Veterinary Research Institute of Chinese Academy of Agricultural Sciences. The rabbit was selected in aging 2 weeks and weighing 180–200 g, without gender restrictions. All the experimental steps followed the guidance of the Ministry of Science and Technology of the People’s Republic of China on caring for experimental animals.

The rabbit was put to death by injecting air into its ear veins and soaked into the 75% alcohol for 20 min. The bilateral femurs of the rabbit were separated at the aseptic ultraclean worktable and dipped in the PBS. The soft tissue of the femurs was removed and instantly soaked in DMEM/F12 medium. Both ends of the femurs were crushed by hemostatic forceps. After that, the bone marrow was flushed out by using a 5-ml syringe containing DMEM/F12 medium for four to five times. The obtained solution was centrifuged at 1200 rpm for 5 min, then the supernatant was removed and the precipitation was kept. The sediment was diluted by adding DMEM/F12 medium containing 13% FBS, 1% penicillin–streptomycin and 1% l-glutamine, and then it was transferred into the aseptic 25-cm^2^ culture flasks for further culture at 37°C and 5% CO_2_ of cell incubator. After 24 h, the flasks were purified with PBS for two or three times and the medium was replaced. When BMSCs attained the top of 80% confluence, the cells were passaged and cultured persistently. The medium was changed every 2–3 days.

The Fb and ECs were purchased from iCell Bioscience Inc., Shanghai, China. The method of Fb culture was consistent with the above, but the culture medium was DMEM/F12 solution supplementing 10% FBS with 1% penicillin–streptomycin and 1% l-glutamine. The culture medium of ECs comprised ECM solution supplementing 5% FBS with 1% the cytokine and 1% penicillin–streptomycin.

### Identification of BMSCs

The coverslips were put into 24-well plate, and the BMSCs were cultured at a density of 2 × 10^4^ cells/cm^2^ with 1-ml culture medium overnight. The solution was aspirated and every well-plate was rinsed with PBS and fixed at 4°C for 30 min with 4% of paraformaldehyde. After that, every well-plate was cleaned with PBS for three to five times. The water in the coverslips was removed and 50-μl blocking solution (composed of 0.5% Triton X-100 mixed with PBS at 1:1 proportion, supplemented 10% FBS) was dropped on every coverslip for 2 h. Briefly speaking, the primary antibodies specific for CD29, CD44 and CD90 were incubated at 4°C, and the tagged secondary antibodies were adopted for 2 h at room temperature in a dark situation. Cell nuclei were stained with DAPI for 5 min. The slides were observed and imaged by the laser scanning confocal microscopy (Olympus FV 1000).

### Cell sheet fabrication

The BMSCs at a density of 9 × 10^4^ cells/cm^2^ were seeded in 6-well plate and cultured for 2 weeks in a high-glucose DMEM medium supplemented with 13% FBS, 1% penicillin–streptomycin, 1% l-glutamine and 50 g/ml ascorbic acid at 37°C in a humidified situation with 5% CO_2_ and after 14 days, the BMCS was obtained. The Fb were seeded in 6-well plate at a density of 5 × 10^4^ cells/cm^2^ and cultured in a high-glucose DMEM medium supplemented with 10% FBS, 1% penicillin–streptomycin and 1% l-glutamine and 0.5 mg/ml ascorbic acid under the above conditions and the Fb cell sheet (FCS) was fabricated after 14 days.

### Pre-vascularized cell sheet fabrication

After 2 weeks, the ECs at a density of 5 × 10^4^ cells/cm^2^ were seeded to the surface of BMCS and FCS were co-cultured for another 5 days. The culture medium was changed every 2–3 days.

### Construction of PTED

After co-cultured for 5 days, according to the methodology by Haraguchi *et al*. [21] with a little modification, 3D of three-layer PTED was contributed by two layers, PBMCS and PFCS, at a sandwich form. Six-layers PTED, folded one time in every cell sheet respectively, was based on the above-mentioned method, and the stacking measure was the same with the above. Three-dimensional PTED, placed in a petri dish mixed with a high-glucose DMEM medium, was supplemented with 13% FBS, 1% penicillin–streptomycin, 1% l-glutamine and 0.5 mg/ml ascorbic acid. The culture medium of ECs, refreshing every 2 days, was incubated at 37°C in a CO_2_ incubator.

### Transplantation of PTED into wound model with nude mice

The animal experiments were carried out according to the approval of the Institute’s Animal Ethics Committee. Forty adult SPF-grade nude male mice (weight, 15–20 g; age, 4–6 weeks) were provided by Beijing Vital River Laboratory Animal Technology Co., Ltd. These animals were kept in the SPF grade of Lanzhou Veterinary Research Institute of the Chinese Academy of Agricultural Sciences. The animals were randomly divided into five groups, respectively, (i) blank control group, (ii) monolayer PBMCS, (iii) monolayer PFCS, (iv) three-layer PTED and (v) six-layer PTED. Among them, the blank control group was regarded as a negative control group; PBMCS and PFCS groups were regarded as positive control groups; three- and six-layer PTED groups were regarded as experimental groups. The nude mice were anesthetized from the abdomen by 10% chloral hydrate (1 ml/250 g). The dorsal of full-thickness skin (1.0 cm × 1.0 cm) was resected on the nude mice. Mono-layer cell sheet and three- and six-layer PTED were cut the same as that of the established model wound by a sterile scalpel. After transplanting PTED into established wounds, the wounds were covered with petroleum jelly gauze, and a set of bandages was used to protect wounds and to prevent displacement or dislocation of the PTED. The wounds were sterilized every other day.

### Evaluation of the wound closure

Wound images were captured via a digital camera (Cannon Inc., Tokyo, Japan) on days 0, 1, 7 and 14 after the wound was healed. By taking photos, the experiments were recorded through a ruler of the same scale. The length and width of the wound were measured on days 1, 7 and 14. The areas of wounds were assessed against scale graph paper (mm^2^). The rates of wound closure were calculated as follows:

$$\frac{10^{2}-\text{Wound}\;\text{area}\;\text{on}\;\text{days}}{10^{2}\times 100\%}$$

The days represent 1st, 7th and 14th day. The wound closure was expressed by mean ± standard deviation (SD) and analyzed by TTEST.

### H&E staining

Collected tissue specimens, fixed in a 10% formalin buffer for 48 h and then dehydrated by graded alcohol series, were derived from the full cross-sectional width of the wound at the 1st, 7th and 14th day after surgery, respectively. Afterwards, they were cleared in xylene and embedded in paraffin. Finally, 3–4 μm thick slices were cut from paraffin blocks and were stained in Hematoxylin and eosin staining (H&E). Histological slides were observed under the optical microscope. The boundary of the wound was distinguished by two blinded examiners who were previously instructed by pathologists.

### Immunofluorescence staining

The PBMCS and the PFCS, washed three times and fixed in a 4% paraformaldehyde solution for 10 min, were purified two to three times after permeabilization with 0.1% TritonX-100 for 5 min and blocked with 1% BSA. The primary antibodies, Rabbit anti-CD31, were incubated for 16 h at 4°C and the tagged secondary antibody, Donkey anti-mouse, was applied for 1.5 h at room temperature in a dark situation. Cell nuclei were stained with DAPI. The slides were observed and imaged by the laser scanning confocal microscopy (Olympus FV 1000).

### Quantification of the density of blood vessels

The number of blood vessels on days 1, 7 and 14 was quantified after CD31 staining. Sections of time points of 1, 7 and 14 days were randomly selected for each group. Finally, the number of blood vessels under each field was viewed as the density of their blood vessels.

### Statistical analysis

All values were performed as mean±SD. Comparisons among the experimental groups, positive control groups and the blank control group, were performed by TTEST in EXCEL to conduct the square-difference analysis. The level of statistical significance was set at *P* < 0.001.

## Results and discussion

### The design concept of PTED and stemness identification of BMSCs

**Figure 1.**
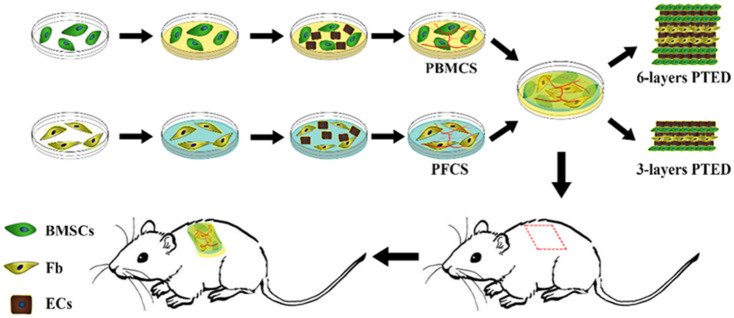
Schematic representation of PTED and animal model construction.

**Figure 2.**
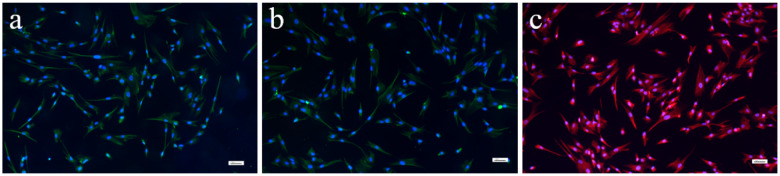
Immunohistochemical staining identified by BMSCs. (**a**) CD29 immunohistochemical staining: green. (**b**) CD44 immunohistochemical staining: green. (**c**) CD90 immunohistochemical staining: red, the nucleus (DAPI staining): blue. Scale bar, 100 μm.

In this study, aiming to mimic the dermis structure and investigate the healing effect of various forms of PTED to dermis repair, we used CST to construct a novel PTED and transplanted it to the dermis-defect model at nude mice. The detailed procedure is shown in Fig. 1. Initially, we created BMCS and FCS, respectively, by using CST, and then they were seeded onto ECs to prepare PBMCS and PFCS. When they were co-cultured *in vitro* for 5 days, the prepared cell sheets were superimposed or folded together, eventually a novel PTED (including three- and six-layer PTED) was constructed. In some literature, it is known that the cultivation of a superimposed three-layer cell sheet on the basis of restoring myocardial function or skin function is conducive to the establishment of the vascular system [22]. Therefore, we chose to build a three-layer PTED. In addition, it has been pointed out that the vascular system gradually extends a number of vascular channels to the surrounding area if it is more than a three-layer cell sheet, which is more conducive to the formation of endogenous blood vessels [23]. In the second place, it has been reported that as the cell sheet continues to stack, the blood vessels rapidly develop layered structures that are easier to construct multi-layered tissue [24]. However, some literature has shown that the superposition of cell sheet of more than 10 layers may lead to the increase of the cell death and the formation of scar tissue owing to the intake of excessive Fb [24]. Then, we finally decided to build six-layer PTED. To evaluate the repair effect, the prepared PTED was transplanted to the dermis-defect model at nude rats. At the given time, the healing effect was quantified by using the methods of histochemistry and immunohistochemistry. The prepared 3D dermis, containing the undifferentiated BMSC and still maintaining the prototype of stem cells, could be used to repair wounds directly and accelerate the healing of wounds. As shown in Fig. 2, immunohistochemical staining analyses of BMSCs showed that they were strongly positive for typical surface antigens, such as CD29, CD44 and CD90. It was confirmed that the cells of the rabbit tibia bone marrow we extracted were indeed BMSCs. Meanwhile, based on the advantages of CST, it can break through the limitations of insufficient donor dermis and immunogenicity of exogenous materials. Besides, Fb, used as the seed cells constructed dermal tissue, which was also the central constituent cells of dermis and the primary repair cells of dermis-defect. As it is known, the shortage of 3D vasculature is the main factor of implantation failure. To resolve this problem, we co-cultured two kinds of different cells to create a pre-vascularized micro-environment [20], which could provide the ample supply of oxygen and nutrition, thus ensuring the survival of cell sheet after they were superimposed or folded together *in vitro*. In this study, PBMCS and PFCS were used to construct PTED, and the existed micro-vessel in the pre-vascularized cell sheet could also promote the formation of vascular networks when PTED was transplanted into the dermis-defect zone in nude rats, finally the formation of 3D vasculature was promoted and the healing of dermis-defect was accelerated.

### Morphotype of PFCS and PBMCS *in vitro*

**Figure 3.**
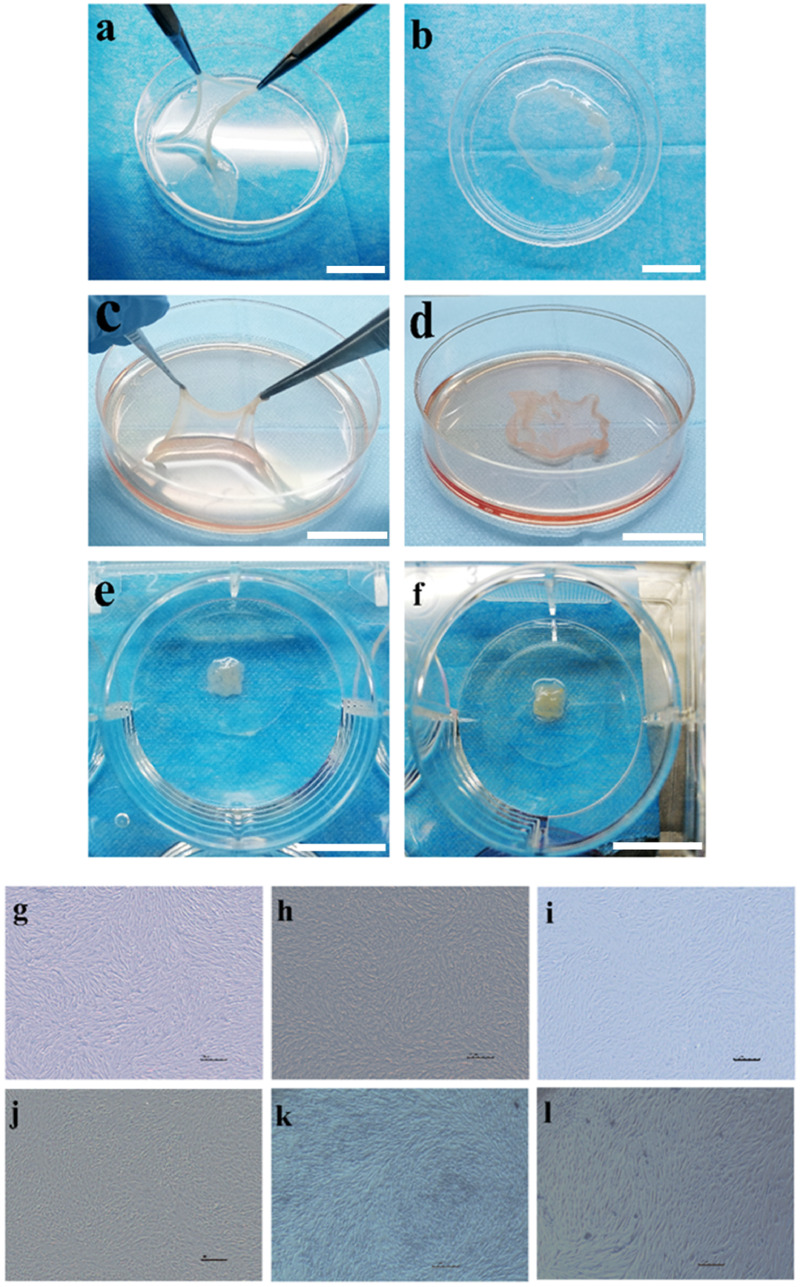
The pre-vascularized cell sheets macrophotograph and BMCS, FCS morphology under the optical microscope. (**a**, **b**) PBMCS. (**c**, **d**) PFCS. (**e**, **f**) Three-layer PTED and six-layer PTED. Scale bar is 1 cm. (**g**, **h**) Incubated FCS at 5th and 10th days. (**i**, **j**) Incubated BMCS at 5th and 10th days. (**k**, **l**) ECs seeded onto FCS and BMCS at 3rd day. Scale bar, 100 μm.

The general view is that PBMCS and PFCS (Fig. 3a–d), incubated about 10–14 days, can grow into milky white and translucent cell sheet at the bottom of the petri dish. Meanwhile, the formed cell sheet can be lifted from the bottom with sterile forceps easily. In addition, we can find that PFCS is thinner than PBMCS. Combing with graphs in an optical microscope, we deduced that the existing ECs might play a key role in arrangements of tightly packed Fb. As shown in Fig. 3e and f, we found that the edge of the cell sheet was curled up, which could be attributed to three reasons: (i) it was that the cell sheet got out of the constraint of the petri dish and became free stretching to release the internal stress. (ii) It was that the cell sheet expressed a large amount of extracellular matrix, including the contractile proteins, facilitating the shrink of the cell sheet. (iii) Finally, it was that the large amounts of cells moving towards the centre of the cell sheet to form a 3D structure, leading to uneven thickness of the cell sheet. In addition to the general view, similar phenomena could be observed under the light microscope. Regardless of the BMCS or FCS, they grew in layers in 5 days under the observation of the inverted microscope (Fig. 3g–j). The cell morphology of BMCS presented a long spindle in 5 days (Fig. 3i). By the 10th day of cell culture, the number of cell layers was more obvious, but the cells lost their typical shape and disorganized, changing from long spindle shape to short spindle and polygon shape (Fig. 3j). For FCS, its cells morphology also showed a long spindle in 5 days (Fig. 3g), like the BMCS, and the cells were arranged in a spiral pattern. When the FCS was cultured to the 10th day, the cell morphology changed to the short spindle, and some cells turned into polygon shape (Fig. 3h), but the change of cell morphology was not as obvious as that of BMCS. When cultivating ECs in the FCS and BMCS, it was surprising to find that significant changes took place for an arrangement of Fb or BMSCs(Fig. 3k and l). In addition, in comparison, the arrangement of Fb was more regular and more spiral shaped. On the whole, it was demonstrated that the ECM of BMCS or FCS acted as a positive influence in the seeded ECs, and vice versa, cultured ECs in both ECM also controlled the cell migration and arrangement.

### Pre-vascularized BMCS and FCS

**Figure 4.**
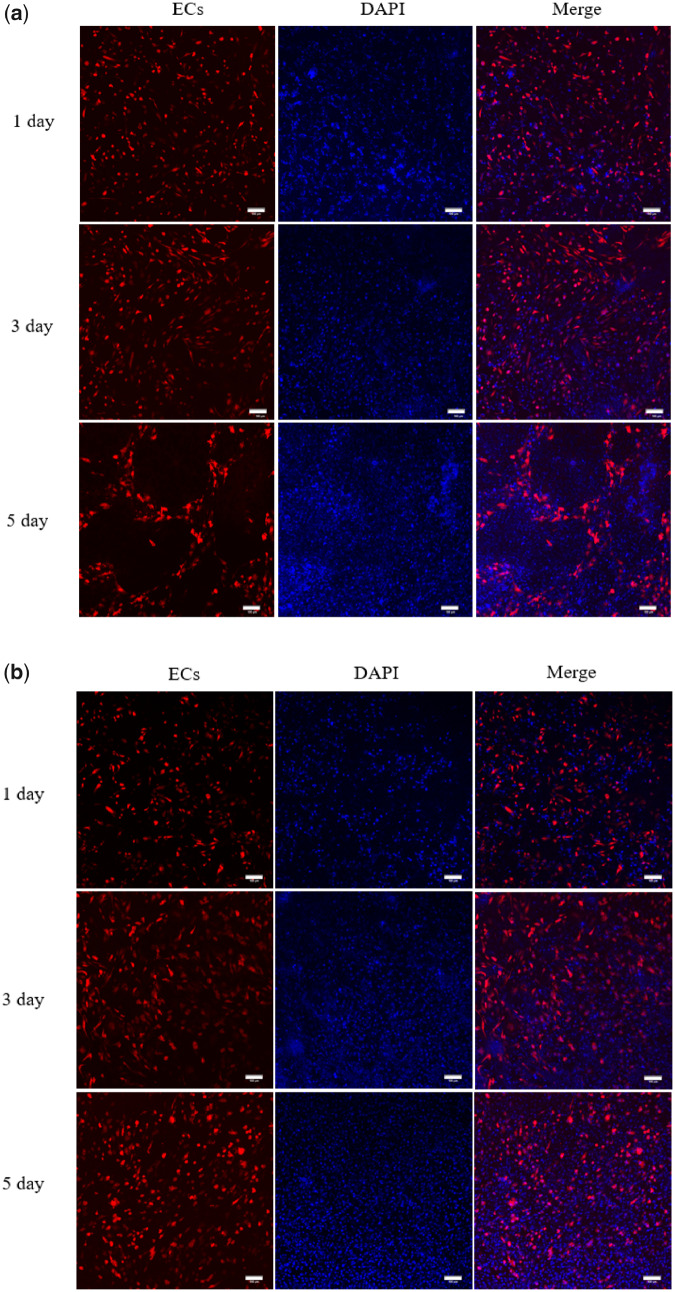
(**a**) PFCS immunofluorescence staining at 1, 3 and 5 days. Blue: the nucleus (DAPI staining) are marked with blue fluorescence. ECs (CD31 staining) are marked with red fluorescence. Scale bar, 100 μm. (**b**) PBMCS immunofluorescence staining at 1, 3 and 5 days. The nucleus (DAPI staining) are marked with blue fluorescence. ECs (CD31 staining) are marked with red fluorescence. Scale bar, 100 μm.

The pre-vascularization effect was quantified through CD31 staining, and the corresponding results are shown in Fig. 4. For PFCS (Fig. 4a), on the first day of co-culture, the distribution of ECs was chaotic and arranged irregularly. On the 3rd day, ECs began to grow spirally and aggregate. On 5th day, the ECs grew into microvessel-like patterns that were similar to the pre-vascularized pattern, further confirming that the co-culture of ECs with Fb was an effective method to promote the pre-vascularization formation [16]. The reason could be that ECM of the FCS acted as an essential role in the pre-vascularized construct, which was possible to induce ECs to express various endothelial growth factors, including Von Willebrand factor, PECAM-1/CD31, VE-cadherin/CD144 and 6 integrin/CDw49f, finally promoting the formation of vascular structure [25–27]. As shown in Fig. 4b, when BMCS were co-cultured with ECs at 1, 3 and 5 days on petri dish surfaces, the changing trend of ECs was not as regular as PFCS group; furthermore, the formed vascular structure was not as obvious as PFCS group. However, it was important for BMCS to promote the pre-vascularization depending on their ECM and secret various signal molecules [28]. In this study, by adopting the method that the multi-potential capacity of differentiation of BMSCs stimulated them to directly differentiate into the various skin cells *in vivo*, the wound healing was finally promoted.

### *In vivo* wound-healing evaluation

**Figure 5.**
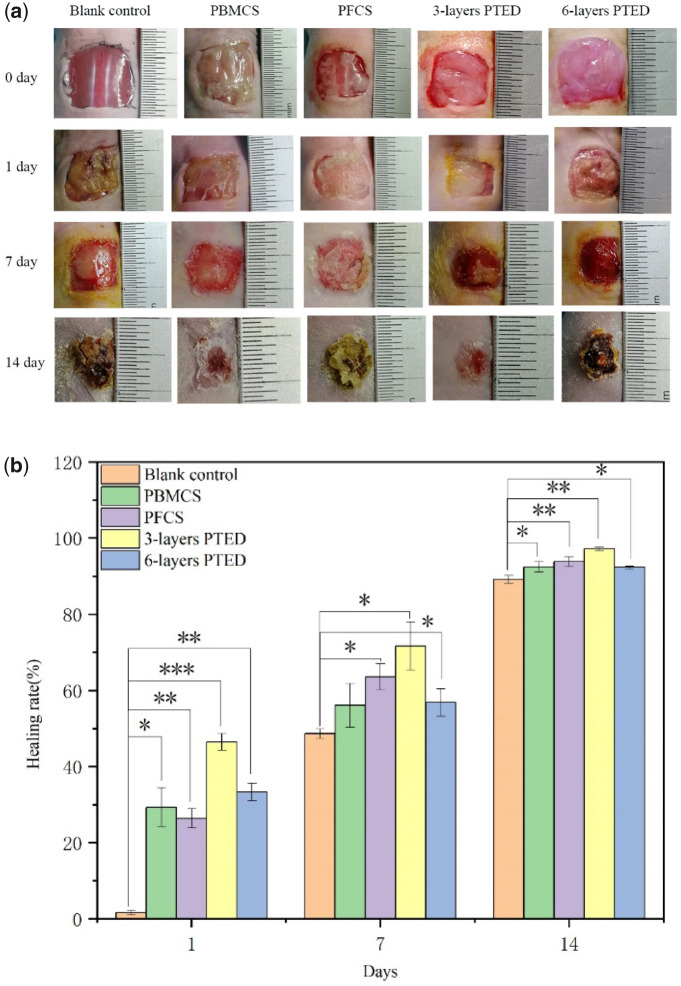
Effects of the different forms of PTED on wound healing. (**a**) Representative macroscopic images of the different conditions at 1, 7 and 14 days. (**b**) Quantification of the percentage of wound-healing rate in the positive control groups, experimental groups and blank control group at 1st, 7th and 14th day of post-operative. All data are presented as mean ± SD; statistically significant is performed as *P < 0.05, **P < 0.01, ***P < 0.001.

**Figure 6.**
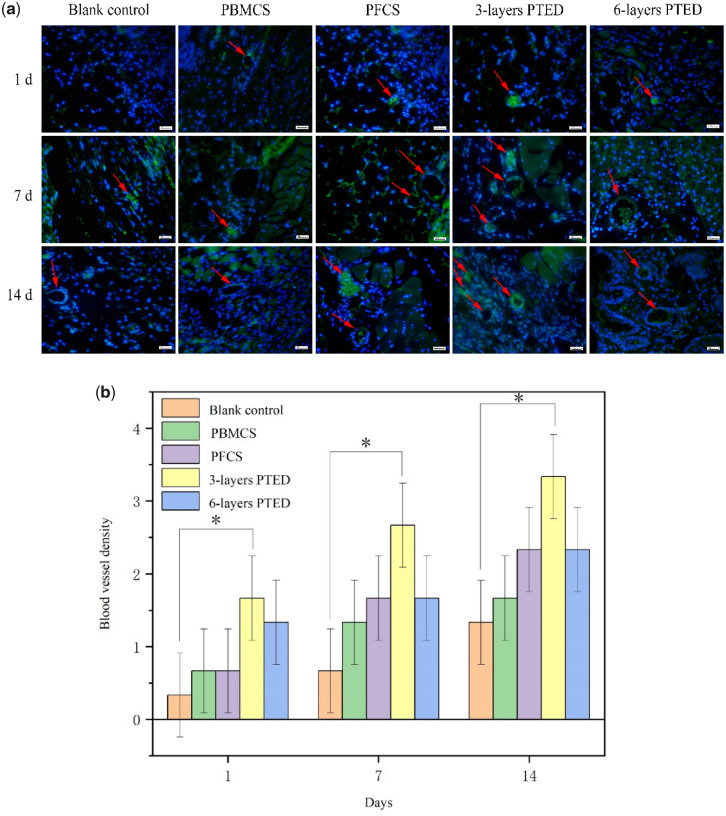
Angiogenesis of blank control group, positive control group and experimental group on day 1, 7 and 14 after surgery. (**a**) Immunofluorescence ECs staining photographs of wound tissue sections in the blank control group, positive control groups and experimental groups at 1st, 7th and 14th days of post-operative. Blue: the nucleus (DAPI), green: the EC (CD31). Scale bar, 100 μm. (**b**) Blood vessel density was quantified in blank control group, positive control group and experimental group at day 1, 7 and 14 after operation. All data are presented as mean±SD (*P < 0.05).

The wound-healing rate was quantified through macroscopic analysis (Fig. 5a) and the wound-healing rate was measured (Fig. 5b) at 1st, 7th and 14th post-operative day. While for the 1st post-operative day, the wound-healing rate of the positive control groups and experimental groups almost reached 30%, which was far higher than the blank control group. In particular, the wound-healing rate in three-layer PTED was greatly higher than the blank control group (*P* < 0.001), and the possible reason might be that the introduction of the cell sheet, containing ample ECM and stem cells, could accelerate the wound healing. As time prolonged, at day 7 the wound-healing rates of positive control groups and experimental groups were higher than that of the blank control group (*P* < 0.05). Among all groups, the wound-healing rate of the three-layer PTED accounted for 71.67%, which was the highest (*P* < 0.05). Surprisingly, the wound-healing rate did not increase with the increase of PTED layers. For six-layer PTED, the wound-healing rate accounted only for 56.8%, which was lower than that of PFCS and three-layer PTED (*P* < 0.05). From day 14 onwards, wounds were progressively closing, especially the three-layer PTED, and the wound-healing rate could reach 97.2%, which indicated that the wound was almost completely healed (*P* < 0.01). For six-layer PTED, the wound healing could reach 92.3%, but this value was almost the same as the positive control groups (*P* < 0.05). Therefore, it could be concluded that three-layer PTED tended to significantly accelerate wound healing, and compared to the other groups, three-layer PTED presented a significant difference in statistics during the whole healing period. Why did the three-layer PTED show the highest wound-healing rate? We speculated that the higher wound-healing rate could be attributed to the following reasons. (i) The composition of PTED, consisting of Fb, BMSCs and ECs, was similar to the dermis. Meanwhile, their cells could secrete a great deal of ECM, such as collagen, fibronectin and laminin, which can directly take part in the healing of wounds [29, 30]. (ii) The pre-formed microvessel-like pattern in the cell sheet played an important role in promoting the formation of 3D vascular networks, which can ensure the survival of PTED when it was grafted to the dermis-defect zone (Fig. 4). (iii) The importance of BMSCs can also not be neglected. Like multi-potential cells, BMSCs can differentiate into various kinds of skin cells, thus promoting the wound healing directly [31]. Compared to the other groups, six-layer PTED, a more massive fibrotic scar appearing as wound healing, can prevent the shrink of wound that might be one of the reasons why this group showed a relatively lower wound healing. Furthermore, the blood vessel density in the six-layer PTED was less than that of the three-layer PTED, which might be the other reason for its lower wound-healing rate (Fig. 6).

### Vascularization effect *in vivo*

Vascularization plays a vital role in promoting the survival of the transplanted PTED and accelerating the dermis-defect wound healing. CD31 is an important mark in evaluating the vascularization effect. Hence, immunofluorescent staining of CD31 was used to evaluate the vascularization effect after wound healing. As shown in Fig. 6, on the first post-operative day, a microvessel-like structure, perhaps coming from the pre-vascularized cell sheets (Fig. 4), could be observed in positive control groups and experimental groups rather than the blank control group. On the 7th day, the microvessel-like structure could be observed in all the groups. It was apparent that the three-layer PTED group had more microvessel-like structures compared to other groups. In the 14th day, it was found that all the groups presented a significant microvessel-like structure. Notably, the PTED group had a higher number of microvessel-like structures in comparison with the other groups. Meanwhile, we can also find that the vascular density of the three-layer PTED group was higher than the six-layer PTED group, which could be the reason why the three-layer PTED group had a higher wound-healing rate (Fig. 5). Furthermore, it has been found that the vessel density of PFCS group was higher than PBMCS group. This result was consistent in the result of the pre-vascularization of the cell sheet (Fig. 4), further verifying that FCS was more suitable than BMCS for promoting pre-vascularization. Above all, we believed that limitations in vascularization and angiogenesis could be server in 3D biological tissue engineering [32]. The oxygen and nutrients, requiring for cell survival, were limited by about 150–200 μm from the blood vessels; therefore, thickness cell sheets may cause hypoxia or the lack of nutrients [33]. As shown in Fig. 5, for the PTED exceeding three-layer PTED, the mechanism of the relationship between the excessive fibrosis of the final wound healing and the blood vessels has not been discussed in detail in this study; hence, a further study is required to be carried out.

### Histological examination

**Figure 7.**
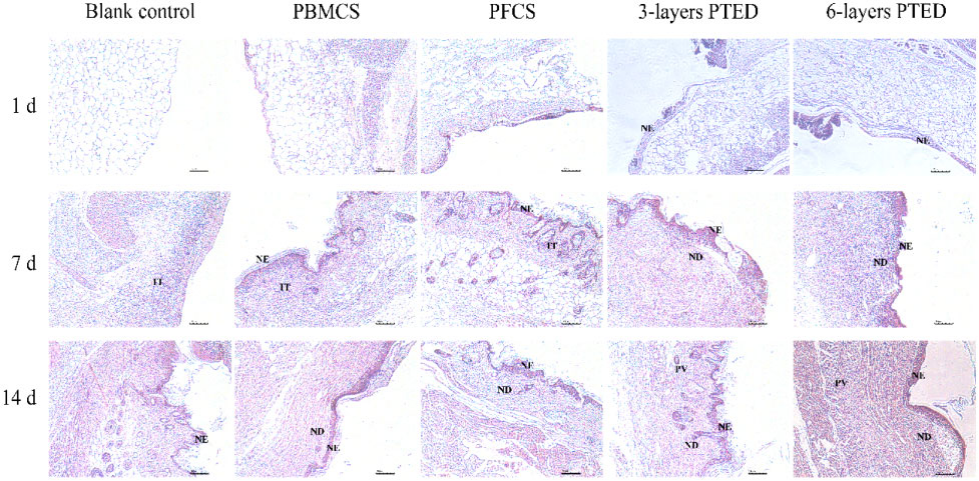
Illustrative images of H&E-stained histological sections of the 1st, 7th and 14th day of post-operative, highlighting the characteristics of wound-healing stages with time. IT, inflammatory tissue; NE, neo-epidermis; ND, neo-dermis; PV, prefabricated vascularized. Scale bar, 100 μm.

Wound-healing analyses, as visualized after H&E staining (Fig. 7), revealed that the beginning of the formation of neo-epidermis (NE), neo-dermis (ND) and pre-fabricated vascularization (PV) at 1st, 7th and 14th day of post-operative could be detected for all wound-healing procedure. On the first day of the wound healing, NE could be detected only in the PTED groups instead of the blank control group, PBMCS and PFCS groups, which meant that PTED groups had a better effect in promoting the formation of NE than the other groups. At day 7, inflammatory tissue (IT) began to arise in the blank control group, PBMCS and PFCS groups, which was more obvious than PTED groups. In addition to the blank control group and PBMCS group, NE tissues were visible in all experimental groups and PFCS group. Compared to the other groups, ND could be observed in PTED groups. The reason may be that the constructed 3D PTED, being a part of dermis tissue and participating in wound healing directly, had a similar composition to the dermis. At day 14, the prefabricated vascularization at the neo-dermis zone was observed in the PTED groups instead of the other groups. Combining with the result of Figs. 5 and 6, it was indicated that compared with the other groups, the pre-vascularization in the PTED groups contributed rapidly to the dermis-defect healing. Meanwhile, we can see that an intact and more thickened NE appeared in the group of three-layer PTED, indicating that this group had a better effect in promoting the formation of NE. The ND being critical in the reconstruction of NE tended to be a possible reason. As ND can provide sufficient mechanical support for NE, it can promote the formation of neonatal vessels based on the ample ECM and the secreted growth factors [34].

At present, although material-based methods are mainly used to repair the dermis defect, there exist some drawbacks, including non-degradability, acidification after degradation, low-cell seeding density and low wound-healing rate [16, 35–43]. Therefore, based on stem-cell technology, including cell injection, cell and gel injection and so on [35, 44], cell-based methods are developed to overcome the drawbacks of material-based methods. However, the limitations of this method include cell loss, decreased cell activity and low wound-healing rate. Compared to the above methods, CST has significant advantages, which can resolve the above- mentioned drawbacks [45, 46]. In the wound healing, mono-layer cell sheet or three-layer cell sheet is used to repair the wound healing [45, 47]. Although a relatively high wound healing rate can be obtained, still a lot of improvement can be made. As it is known, pre-vascularization plays a vital role in sustaining the survival of the transplanted tissues and accelerating wound healing. Therefore, we tried to construct the PTED in this study, aiming to improve the healing effect based on CST. As far as we know, there are few reports on the application of cell sheets to wound healing, let the application of PTED alone be the one to repair dermis-defect. The advantages of this strategy can be summarized as follows. (i) The pre-vascularized cell sheet plays an essential role in sustaining the survival of PTED *in vitro* and PTED transplanting into the nude rats. On the one hand, the pre-vascularized cell sheet can ensure the rapid formation of microvessels between the layer and the layer of the cell sheet. In addition, the pre-fabricated microvessel in the PTED anastomoses with the host vascular, thus promoting the formation of the 3D vascular network. Therefore, this method can be the effective remedy for the shortage of 3D vascular network in the process of tissue implantation. More importantly, the introduction of microvessels can accelerate wound healing. (ii) The constructed PTED is mainly composed of BMSCs, Fb, ECs and their secreted ECM, and hence the constituent of PTED is similar to the real structure of the dermis. As a part of the dermis, the prepared PTED can directly take part in the process of wound healing, which may be one reason in shortening the wound healing time. (iii) The cells used for cell sheet construction can be obtained from the patients so that the immunological rejection reaction can be effectively avoided. Herein, another limitation of insufficient donor dermis can also be resolved. (iv) The BMSCs in PTED can differentiate into various skin cells, such as Fb, ECs and keratinocyte, which can provide various seed cells for wound healing [42]. (v) Different from the previously reported methods, PTED can be viewed as a part of the dermis and directly take part in the wound healing, and hence PTED does not need to be removed after the wound healing. With the above-mentioned advantages, the three-layer PTED underlined a faster wound-healing rate compared to the most reported methods, such as nanomaterials, hydrogels, stem cells and mono-layer cell sheet, and even commercial wound dressing (Tegaderm™)[16, 32–40, 48]. In this study, the wound-healing rate of six-layer PTED was lower than three-layer PTED, and the specific reasons require further study.

## Conclusion

To solve the problems of insufficient donor dermis and the shortage of 3D vasculature, novel PTED was developed based on CTS. The constructed PTED had a similar cell composition with the dermis, including ECs, BMSCs and Fb. More importantly, the constructed PTED had microvessel-like structures that enabled the survival of the transplanted PTED and the quick wound healing *in vivo*. The wound-healing rate of three-layer PTED could reach 97.2% in 14th post-operative day, which was both higher than the other groups and most of the reported materials owing to its higher number of functional vascular formation. In conclusion, the PTED not only provides a new treatment method for the dermal defect of the skin but also expands the application scope of cell sheets in tissue engineering.

## Funding

This study is supported by The Natural Science Foundation of China (81571829), The Fundamental Research Funds for the Central Universities (lzujbky-2020-it29) and the open project of State Key Laboratory of Solid Lubrication, Lanzhou Institute of Chemical Physics, Chinese Academy of Sciences (LSL-1907). 

## Conflict of interest

None declared. 
